# Supplementary material for: Do Urban Hedgehogs (Erinaceus europaeus) Represent a Relevant Source of Zoonotic Diseases?
Source: Pathogens. 2023 Feb 7;12(2):268. doi: 10.3390/pathogens12020268 (PMC9961789; doi:10.3390/pathogens12020268)
Supplement: Supplementary file 1 [file pathogens-12-00268-s001.zip › pathogens-2194477-supplementary.pdf]

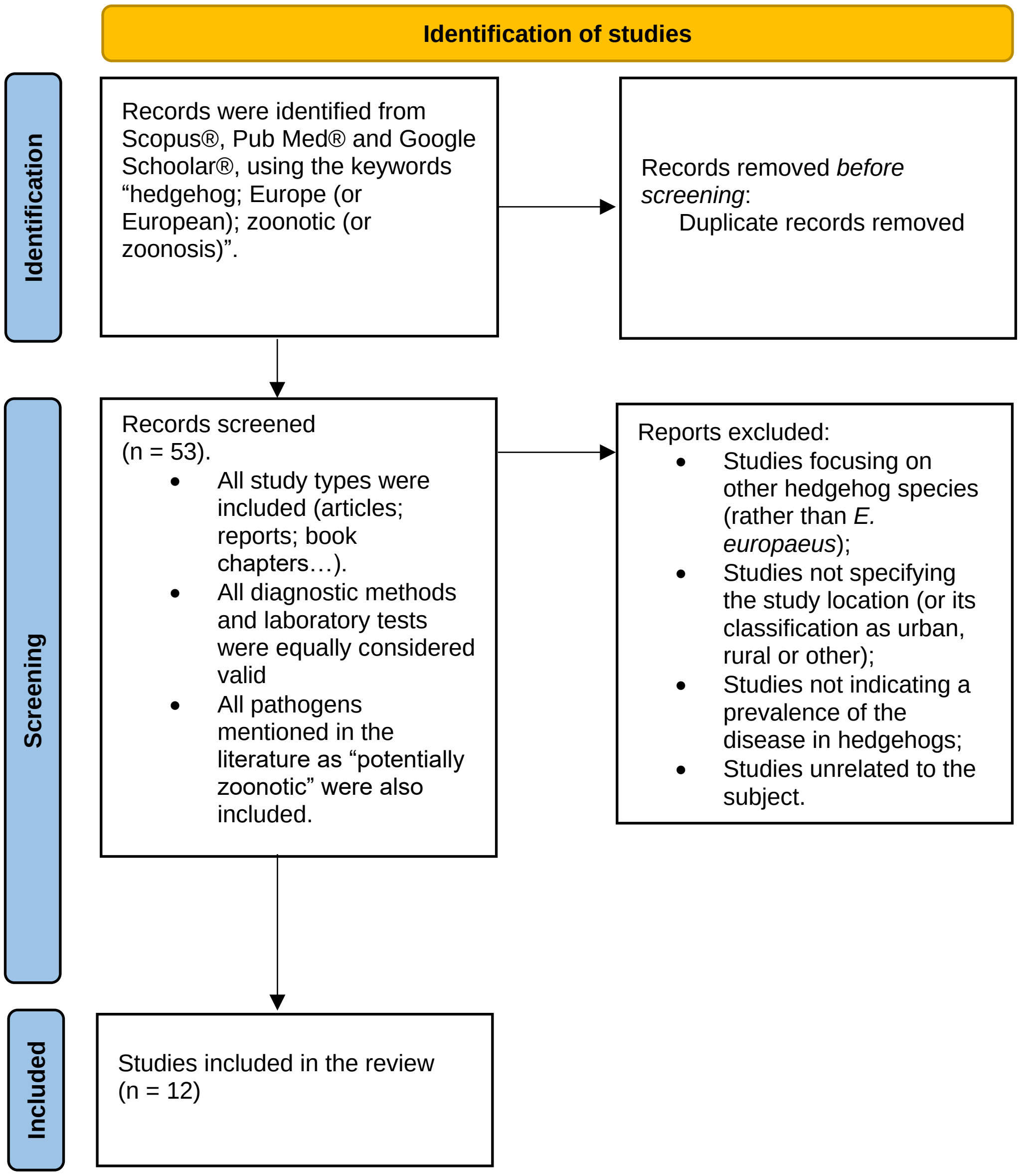

Figure S1: Flow diagram for “Do urban hedgehogs (Erinaceus europaeus) represent a relevant source of zoonotic diseases?”

Diagram designed based on: Page MJ, McKenzie JE, Bossuyt PM, Boutron I, Hoffmann TC, Mulrow CD, et al. The PRISMA 2020 statement: an updated guideline for reporting systematic reviews. BMJ 2021;372:n71. doi: 10.1136/bmj.n71
